# The Impact of a Six-Hour Light–Dark Cycle on Wheat Ear Emergence, Grain Yield, and Flour Quality in Future Plant-Growing Systems

**DOI:** 10.3390/foods13050750

**Published:** 2024-02-28

**Authors:** Helena Clauw, Hans Van de Put, Abderahman Sghaier, Trui Kerkaert, Els Debonne, Mia Eeckhout, Kathy Steppe

**Affiliations:** 1 Laboratory of Plant Ecology, Department of Plants and Crops, Faculty of Bioscience Engineering, Ghent University, Coupure Links 653, 9000 Ghent, Belgium; helena.clauw@ugent.be (H.C.); hans_vandeput@hotmail.com (H.V.d.P.); abderahman.sghaier@ugent.be (A.S.); trui.kerkaert@gmail.com (T.K.); 2 Research Unit of Cereal and Feed Technology, Department of Food Technology, Safety and Health, Faculty of Bioscience Engineering, Ghent University, Valentin Vaerwyckweg 1, 9000 Ghent, Belgium; els.debonne@ugent.be (E.D.); mia.eeckhout@ugent.be (M.E.)

**Keywords:** spring wheat, *Triticum aestivum*, photoperiod, space, closed environment, vertical farming

## Abstract

Cultivating wheat (*Triticum aestivum*) in a closed environment offers applications in both indoor farming and in outer-space farming. Tailoring the photoperiod holds potential to shorten the growth cycle, thereby increasing the annual number of cycles. As wheat is a long-day plant, a night shorter than a critical length is required to induce flowering. In growth chambers, experiments were conducted to examine the impact of a 6 h light–dark cycle on the timing of wheat ear emergence, grain yield, and flour quality. Under equal daily light-integral conditions, the 6 h light–dark cycle promoted growth and development, resulting in accelerated ear emergence when compared to a 12 h cycle, additionally indicating that 12 h of darkness was excessive. To further stimulate heading and increase yield, the 6 h cycle was changed at the onset of stem elongation to a 14 h–10 h, mimicking spring conditions, and maintained until maturity. This successful transition was then combined with two levels of light intensity and nutrient solution, which did not significantly impact yield, while tillering and grain ripening did increase under higher light intensities. Moreover, it enabled manipulation of the baking quality, although lower-end falling numbers were observed. In conclusion, combining a 6 h light–dark cycle until stem elongation with a 14 h–10 h cycle presents a promising strategy for increasing future wheat production in closed environments. The observation of low falling numbers underscores the importance of factoring in flour quality when designing the wheat-growing systems of the future.

## 1. Introduction

Current research on wheat (*Triticum aestivum*) growth in a closed environment considers both indoor farming [1] and space applications [2,3,4]. The key focus in both applications is maximizing yield while minimizing energy input. Accelerating the growth cycle increases cumulative annual yield by allowing multiple harvests per year [1] Under the right conditions, including a long photoperiod, a 70-day growth cycle from sowing to maturity can be reached, which implies five complete growth cycles in one year [5]. Besides a long photoperiod, temperatures of over 22 °C and high plant densities of over 700 plants per m^2^ are also frequently used in research on indoor wheat cultivation [2,3,5,6]. Based on the results of Monje and Bugbee [5], Asseng et al. [1] calculated an annual wheat yield of 700 ± 40 t ha^−1^ in a ten-layer indoor vertical facility, which corresponded to 220 times the current world average of annual wheat yield of 3.2 t ha^−1^. Under optimised conditions, a theoretical maximum annual wheat yield of even 1940 ± 230 t ha^−1^ was estimated. According to Asseng et al. [1], given current market prices, indoor wheat farming is still not expected to be economically feasible soon.

A shortening of the growth cycle duration by increasing the photoperiod is often accompanied by a reduction in yield [7], indicating a complex trade-off between growth cycle duration and yield. Since lighting is the most important share of energy consumption for indoor wheat cultivation, the optimisation of lighting is one of the main ways of improving energy efficiency [7]. Apart from the photoperiod, light intensity also has an important effect on both yield and growth cycle duration [8,9]. Dong et al. [2] showed the possibility of lowering light intensity during the seedling stage (first 20 days) without any effect on yield, further adding to the complexity of improving yield while minimizing energy input. In addition to the photoperiod and light intensity, light quality also plays an important role in yield optimisation [9,10]. In addition to grain yield, the quality of both the grain and the resulting flour, including baking and nutritional aspects, is of great importance. While some research has already explored the baking and nutritional quality of wheat grown in a closed environment [2,3,7,9], research on this topic remains limited. Since α-amylase is responsible for starch degradation, α-amylase activity is considered an important indicator for end-product quality in the baking industry [11,12]. The falling number is frequently used to quantify α-amylase activity, with a low falling number reflecting a high level of α-amylase activity [13]. Although low falling numbers (below 250–350) currently lead to the rejection of grains for bread baking, recent evidence suggests that the two main causes for a low falling number, late-maturity α-amylase and pre-harvest sprouting, have different effects on end-product quality [11,12]. Besides the falling number, several other parameters can be used to describe and evaluate baking quality, like Mixolab dough properties, Zeleny sedimentation number, and gluten and protein content [14].

While some research has already been conducted on understanding the effect of photoperiod on spring wheat grown in a closed environment (e.g., [7,15]), previous studies have been limited to the 24 h light–dark cycle of the Earth, where one period of light is followed by one period of darkness. In our study, the goal was to go one step further by incorporating multiple periods of light and darkness within a 24-h day. We therefore investigated and compared the impact of a 12 h light–dark cycle (as previously described by, e.g., Evtushenko and Chekurov [8]) with two cycles of 6 h of light followed by 6 h of darkness in one day, ensuring that both treatments had equal daily light-integral conditions. Specifically, we investigated and compared their effects on ear emergence in spring wheat (*Triticum aestivum* ‘Servus’). As spring wheat is a long-day plant that requires a period of darkness shorter than a critical night length to induce flowering, the 6 h period of darkness is anticipated to shorten the vegetative phase, potentially boosting wheat production in future plant-growing systems. Additionally, we investigated whether the 6 h light–dark cycle persisted into the generative phase by comparing plant development, grain yield, and nutritional and baking quality under two conditions: the 6 h cycle maintained until maturity, and a transition from this cycle to a 14 h–10 h light–dark cycle, mimicking spring conditions in the field (e.g., Monostori et al., 2018 [9]), upon the onset of stem elongation. We also examined two levels of light intensity and nutrient solution.

## 2. Materials and Methods

### 2.1. Experimental Set-Up

Three experiments were conducted on spring wheat (*Triticum aestivum* L. ‘Servus’), grown on a lava-rock substrate. This substrate was chosen with application on Mars in mind, where either Mars rocks or regenerated biochar could be used as substrate. In our research project, we required a spring wheat cultivar, with no vernalisation need and therefore a shorter growth cycle than winter wheat cultivars. After careful consideration, we selected ‘Servus’ due to its widespread availability and excellent baking quality. This choice was paramount to ensure consistency and reliability in our experiments, as ‘Servus’ not only met our criteria for accessibility but also offered desirable traits crucial for our investigation. Before sowing, seeds were disinfected by immersing them in 70% ethanol for two minutes and in 20° household bleach (sodium hypochlorite) (Lambert Chemicals SPRL, Herstal, Belgium) for ten minutes, after which the seeds were rinsed five times with deionised water. 

The first (EXP1) and second (EXP2) experiments were both conducted in two growth chambers at the Faculty of Bioscience Engineering at Ghent University, Belgium (51°3′ N, 3°42′ E). Each chamber mimicked a future plant-growing system that could be deployed on Mars and was equipped with a tray containing 180 small baskets filled with lava-rock substrate constantly submersed in Hoagland nutrient solution. Nutrient solution was added two times per day for five to ten minutes to compensate for evapotranspiration. This approach aimed to maintain the stability of the solution’s electrical conductivity (EC), a factor discussed in further detail below. Overflowing water was captured at the opposite end of the table. Initially, three seeds were sown per basket, after which the surplus of germinated and non-germinated seeds was removed, resulting in a plant density of 275 plants per m^2^. 

EXP1 was conducted in 2021 from day of year (DOY) 8 to DOY139. A different light–dark cycle was used in each growth chamber. In contrast to prior studies that investigated only one cycle of light followed by darkness, our aim was to introduce multiple cycles of both light and darkness within a 24 h day. To facilitate comparison, we ensured that both treatments received equal amounts of light over a 24 h period, thereby ensuring they experienced equal daily light-integral conditions. We decided to test a 6 h light–dark cycle, in which a 6 h period of light was followed by a 6 h period of darkness. To ensure an equal daily light integral, the second growth chamber was programmed with a 12 h light–dark cycle, consisting of a 12 h period of light followed by a 12 h period of darkness. This latter photoperiod was previously also tested by Evtushenko and Chekurov [8]. Lighting was provided by prototype LED lamps (Rosa fixture, RAYN Growing Systems—ETC, London, UK) at an initial distance of 90 cm above the tray. At table level, the light intensity was about 93 µmol m^−2^ s^−1^. The light spectrum consisted of 16.9% blue (400–500 nm), 14.6% green and yellow (500–600 nm), 54.6% red (600–700 nm) and 13.9% far-red (700–800 nm) light (Figure S1). To deal with plant elongation, lamps were raised on DOY111 or 103 days after sowing (DAS) to 100 cm for the 6 h treatment. For the 12 h treatment, lamps were raised to 108 cm and 119 cm on DOY86 (78 DAS) and DOY111 (103 DAS), respectively. Trays were flushed once a week to keep the EC and pH as constant as possible (Table 1). The EC and pH of the nutrient solution on the table were measured about once a week using a portable pH/EC/DO meter (HI98199, Hanna Instruments, Temse, Belgium). In both growth chambers, day and night temperatures, relative humidity, and atmospheric CO_2_ concentration were set to equal values and kept constant (Table 1). Microclimate was continuously monitored. Photosynthetic active radiation (PAR) and CO_2_ concentration were measured with an LI-190 Quantum Sensor (LI-COR, Lincoln, NE, USA) and a GMP252 carbon dioxide probe (Vaisala CARBOCAP, Vantaa, Finland), respectively. Air temperature and relative humidity were measured with a temperature and relative humidity sensor (SHT25, Sensirion AG, Stäfa, Switzerland), installed in a ventilated radiation shield. All microclimate measurements are summarised in Table 1. For the 6 h treatment, grain harvest started at 131 DAS. Since ears only started to emerge 199 DAS in the 12 h treatment, pointing to a night that likely exceeded the critical length, no grains were harvested in this treatment.

EXP2 was conducted in 2022 from DOY17 to DOY144 at the same location as EXP1. Building upon the success of applying the 6 h light–dark cycle in EXP1 and because spring wheat is a long-day plant, our objective was to investigate whether the 6 h light–dark cycle persisted into the generative phase until maturity or if transitioning to a 14 h–10 h light–dark cycle, mimicking spring conditions (e.g., [9]), around the onset of stem elongation would yield greater benefits. Until the beginning of stem elongation, a 6 h light–dark cycle was therefore used in both treatments. Around the beginning of stem elongation, which was visually determined on the growing plants, the light regime in one treatment was transitioned to a longer photoperiod of 14 h followed by a 10 h period of darkness, aiming to shorten the duration of stem elongation, and thus the period until heading [16]. For simplicity, this treatment is referred to as the 14 h–10 h treatment, but it is noteworthy that during the initial 37 days, these wheat plants were subjected to the 6 h light–dark cycle. The other treatment, in which the 6 h cycle was maintained until maturity, is referred to as the 6 h treatment. Five Alina lamps (RAYN Growing Systems—ETC, London, UK) per growth chamber were used to provide uniform lighting conditions. Lamps were raised regularly to maintain at all times a distance of 50 to 70 cm above the plants’ canopy. At canopy level, the light intensity was 440–720 µmol m^−2^ s^−1^, and the light spectrum consisted of 12.3% blue, 32.0% green and yellow, 40.9% red, and 14.8% far-red light (Figure S1). In contrast to EXP1, the nutrient solution’s pH was lowered to 6 by adding nitric acid. Additionally, oxygen was actively added through a venturi injector to maintain a high dissolved oxygen (DO) concentration. Trays were flushed three times a week to keep the EC, pH, and DO constant (Table 1). EC, pH, and DO values of the nutrient solution on the table were measured every two to three days. These values were measured directly before and after adding new nutrient solution to the system (Table S1). After adding the new nutrient solution, we observed no significant changes in EC levels in both treatments, indicating the success of our approach. For the 6 h cycle, no significant changes in pH were observed; this contrasts with the results of the 14 h–10 h cycle. However, the *p*-values were close to the 0.05 level of significance, being 0.07 and 0.04, respectively. For DO of the nutrient solution, significant changes after adding new nutrient solution were observed as a result of the venturi injector. Microclimate was continuously monitored with the same sensors as described for EXP1 (Table 1). For the 6 h and 14 h–10 h treatments, harvest started at 122 and 127 DAS, respectively. 

The third experiment (EXP3) was conducted in a growth chamber at Urban Crop Solutions, Waregem, Belgium (50°52′ N, 3°19′ E) in 2022 from DOY75 to DOY186. Eight growth tables were covered with a cotton-fibre mat on which two plastic trays with 54 baskets filled with lava-rock substrate were placed. Again, three seeds were sown per basket and the surplus of seeds was removed, resulting in a plant density of 225 plants per m^2^. Drawing from the results obtained in EXP2, in all treatments in EXP3, a 6 h light–dark cycle was initially adopted before stem elongation. This regime was then transitioned to a 14 h–10 h light–dark cycle after the onset of stem elongation. Lighting was provided by a combination of white, blue, red, and far-red LEDs. For all treatments, lamps were installed on nonadjustable fixtures at a height of 79 cm above the trays. The light spectrum consisted of 11.7% blue, 18.8% green and yellow, 52.0% red, and 17.4% far-red light (Figure S1). Two different light intensities were used: a low light intensity of about 300 µmol m^−2^ s^−1^ (LL) at tray level, and a high light intensity of about 500 µmol m^−2^ s^−1^ (HL) at tray level. These light intensities were applied in combination with two nutrient solution concentrations: a nutrient solution with a high EC of 2000 µS cm^−1^ (HN), and one with a low EC of 1500 µS cm^−1^ (LN) (Table 1). This resulted in four different treatments: HL + HN, HL + LN, LL + HN, and LL + LN. In contrast to EXP1 and EXP2, an ebb-and-flow system with a duration of seven minutes and a frequency of one hour was used for EXP3. Once per week, nutrient solution coming from the automatic watering system was captured and EC, pH, and DO values were measured. In this set-up, it was impossible to measure nutrient solution directly on the table because of the used substrate. After 51 days, the light intensity of the HL treatments was lowered to the same intensity as that of the LL treatments to prevent too-high light intensities at ear level. The EC of all treatments was lowered to 1000 µS cm^−1^ 68 DAS and to 500 µS cm^−1^ 98 DAS to adapt the nutrient supply to the plant’s needs at different developmental stages [4]. To test whether reducing the EC improved the senescence of the leaves, the greenness of flag leaves was measured using the SPAD 502 Plus Chlorophyll Meter (Spectrum Technologies, Aurora, IL, USA) on four selected plants at 68 (*n* = 4) and 97 (*n* = 4) DAS, and on different plants, used for harvest, at 113 DAS (*n* = 20). Microclimate was continuously measured with two sets of the same sensors as in EXP1 and EXP2 (Table 1). For all treatments, harvest started 111 DAS.

### 2.2. Harvest

During harvest, the total number of grains per ear and the total fresh and dry weights of all grains per ear were determined on plants of the 6 h (*n* = 38) treatment in EXP1, the 6 h (*n* = 79) and 14 h–10 h (*n* = 66) treatments in EXP2, and the HL + HN (*n* = 20), HL + LN (*n* = 21), LL + HN (*n* =20), and LL + LN (*n* = 20) treatments in EXP3. The numbers of shoots with and without grains were counted in EXP2 and EXP3, while only the number of shoots with grains was determined in EXP1. These measurements were performed on plants of the 6 h (*n* = 38) treatment in EXP1, the 6 h (*n* = 102) and 14 h–10 h (*n* = 76) treatments in EXP2, and the HL + HN (*n* = 19), HL + LN (*n* = 21), LL + HN (*n* = 20), and LL + LN (*n* = 19) treatments in EXP3.

Based on these measurements, the following characteristics were determined per plant and compared within experiments: total dry weight of all grains per plant, average number of grains per ear, dry weight per grain, and average dry matter content per grain. Statistical analysis was performed using R [17] in RStudio [18]. For visualisation, the ‘ggplot2’ package was used [19]. Treatments in EXP2 were compared using the non-parametric Kruskal–Wallis test, because either non-normality, heterogeneity of variances, or both were observed. In EXP3, the effects of light intensity and EC on the same characteristics were assessed using a linear regression model after normality and homogeneity of variances were checked. The effect of the light–dark cycle on the total number of shoots per plant and number of shoots with grains per plant in EXP2 was assessed using a quasi-Poisson regression model. In EXP3, the effect of light intensity and nutrient concentration on the total number of shoots per plant and number of shoots with grains per plant was also assessed using a quasi-Poisson regression model. Differences between the mean values of each treatment were tested for all possible combinations in EXP3, using a post hoc pairwise comparison from the ‘emmeans’ package [20] (Lenth, 2023), for both the linear and quasi-Poisson regression models. 

Total potential grain yield per m^2^ was estimated based on the measured dry weight of all grains per plant, plant density, and a dry matter content of 85%. Additionally, the dry matter content of all grains per shoot was determined based on measurements of the dry and fresh weights of all grains per ear. 

### 2.3. Quality Analysis

In EXP3, a complete quality analysis was performed on ripe grains. In EXP2, insufficient material was obtained, precluding the measurement of most analysed quality parameters. Only parameters feasible for measurement were analysed. EXP1 lacked sufficient harvested material for a quality analysis to be performed. 

Prior to milling, kernel characteristics, including moisture content (MC, g 100 g^−1^) and the yielding parameters thousand kernel weight (TKW, g) and test weight (TW, kg hL^−1^ or weight in kg per 100 L of kernels), were determined. A Dickey-John apparatus was used to simultaneously measure the MC and TW on two replicates of the same light and EC treatment batch. TKW was determined, for EXP2 and EXP3, by weighing 4 × 100 kernels counted with a manual seed counter. 

Wholemeal flour was obtained using a FOSS Hammertec mill (FOSS, Hilleroed, Denmark). The hammer mill was equipped with a 0.8 mm sieve and was certified by the AACC, ICC, and ISO standards to be suitable for use as a sample mill for analysis of Hagberg falling numbers. After milling, wholemeal flour samples were stored in high-density polyethylene containers until further analysis. Dry matter (g kg^−1^) of the wholemeal flour was determined following ISO 6496. Mineral content (mg kg^−1^) was analysed using the ICP-AES method (ISO 11885): boron (B), calcium (Ca), copper (Cu), iron (Fe), potassium (K), magnesium (Mg), manganese (Mn), sodium (Na), phosphorus (P), and zinc (Zn). The Hagberg falling number was determined using a Perten falling number of 1310 (ICC 107/1) as a measure for the α-amylase activity in the wholemeal flour. Crude protein content was determined using the Dumas method (ISO 16634-1; VarioMax C/N, Elementar Analysesystemen, Langenselbold, Germany; conversion factor 5.7). Gluten quality was measured with a Glutomatic (wet gluten, dry gluten, gluten index (GI); AACC 38-12.02) on the wholemeal flour. Except for the Hagberg falling number and gluten quality, these parameters were also measured in EXP2. 

A Mixolab was used to study the rheological and enzymatic behaviour of doughs in combination with temperature changes and mixing of the dough. The AACC 54-60.01 was used to define the following dough characteristics: C1, C2, C3, C4, C5, α, β, and γ. Based on the Mixolab curve, C1 presents the maximal value of torque (Nm) at the first stage of mixing and is used to determine water absorption, C2 gives an indication of protein weakening in terms of mechanical energy and temperature, C3 measures the starch gelatinisation and peak viscosity, C4 measures the stability of the gel and the α-amylase activity in the dough, and C5 measures starch retrogradation in the cooling stage and final viscosity. 

Quality parameters were compared using a non-parametric Kruskal–Wallis test.

## 3. Results

**Figure 1:**
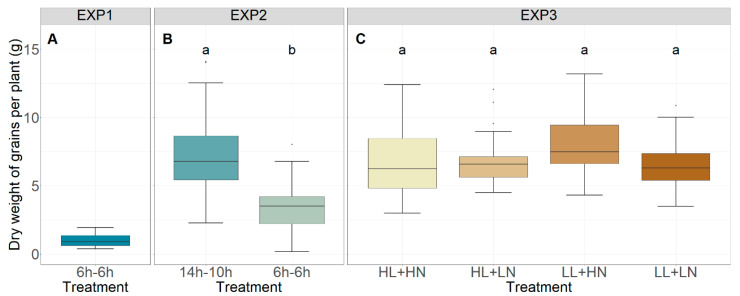
Total dry weight of grains per plant in EXP1 (**A**), EXP2 (**B**), and EXP3 (**C**). HL, LL, HN, and LN refer to ‘high light intensity’, ‘low light intensity’, ‘high nutrient concentration’, and ‘low nutrient concentration’, respectively. Different letters indicate statistical differences (*p* < 0.05) within the experiments.

**Figure 2:**
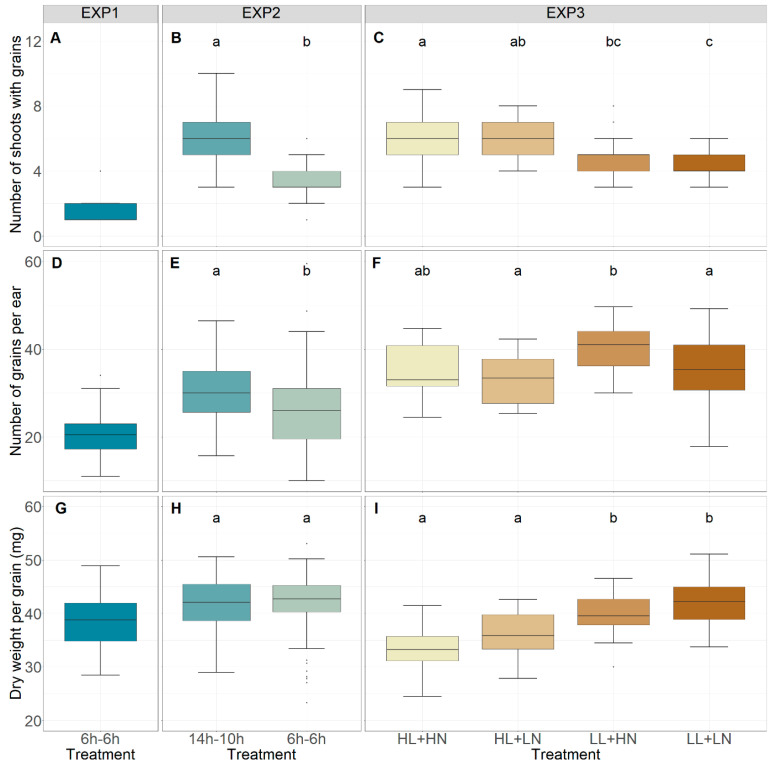
Number of shoots with grains in EXP1 (**A**), EXP2 (**B**), and EXP3 (**C**); number of grains per ear for EXP1 (**D**), EXP2 (**E**), and EXP3 (**F**); and dry weight per grain (g) for EXP1 (**G**), EXP2 (**H**), and EXP3 (**I**). HL, LL, HN, and LN refer to ‘high light intensity’, ‘low light intensity’, ‘high nutrient concentration’, and ‘low nutrient concentration’, respectively. Different letters indicate statistical differences (*p* < 0.05) within the experiments.

**Figure 3:**
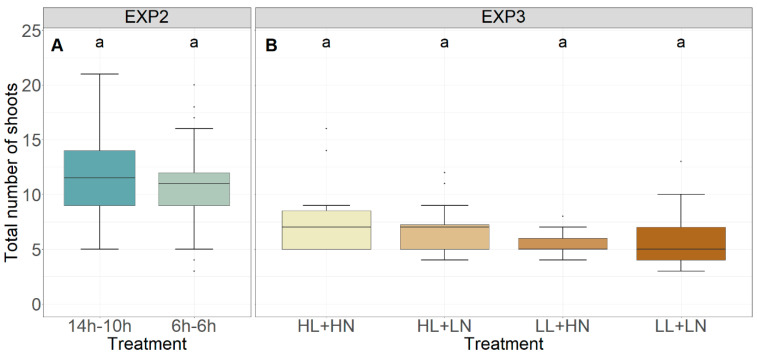
Total number of shoots for EXP2 (**A**) and EXP3 (**B**). HL, LL, HN, and LN refer to ‘high light intensity’, ‘low light intensity’, ‘high nutrient concentration’, and ‘low nutrient concentration’, respectively. Different letters indicate statistical differences (*p* < 0.05) within the experiments.

The total dry weight of all grains per plant for each experiment is given in Figure 1. The number of shoots with grains, number of grains per ear, and dry weight per grain are displayed in Figure 2, and the total number of shoots is given in Figure 3. In EXP2, the total dry weight of all grains per plant was significantly higher for the 14 h–10 h treatment, which received a 6 h light–dark cycle during the initial 37 days (start of stem elongation), compared to the 6 h treatment, which maintained the 6 h light–dark cycle until maturity (*p* < 0.001, Figure 1B). A more detailed analysis of all components that determine the total dry weight showed that the number of shoots with grains (Figure 2B) and the number of grains per ear (Figure 2E) were significantly higher in the 14 h–10 h treatment (*p* < 0.001), while no significant effect for the dry weight per grain (*p* > 0.05, Figure 2H) was observed. No significant difference in the total number of shoots per plant was observed between the 14 h–10 h and 6 h treatments in EXP2 (*p* > 0.05, Figure 3A). 

In EXP3, the total dry weight of grains per plant was not significantly affected by light intensity or nutrient concentration in plants that were initially grown under a 6 h light–dark cycle until stem elongation and then transitioned to the 14 h–10 h light–dark cycle until maturity (*p* > 0.05; Figure 1C). The number of grains per ear (Figure 2F) and dry weight per grain (Figure 2I), however, increased under low light intensity (*p* < 0.001 and *p* < 0.05, respectively), while decreases in the number of shoots with grains (*p* < 0.01, Figure 2C) and the total number of shoots (*p* < 0.05, Figure 3B) were observed. Neither the number of shoots with grains (Figure 2C), number of grains per ear (Figure 2F), dry weight per grain, (Figure 2I) nor the total number of shoots (Figure 3B) were significantly affected by nutrient solution (*p* > 0.05). No significant effect of interaction between the light intensity and nutrient concentration treatments was observed (*p* > 0.05). 

The timing of ear emergence for each experiment is summarized in Table 2. Ears had already started to emerge 66 DAS for the 6 h treatment in EXP1, while this took 199 DAS for the 12 h treatment. In EXP2, the start of ear emergence was further reduced to 60 and 56 days for, respectively, the 6 h and 14 h–10 h treatments. In EXP3, ear emergence only took 51 to 54 days. Ear emergence was always closely followed by flowering, with a maximum delay of one week.

**Figure 4:**
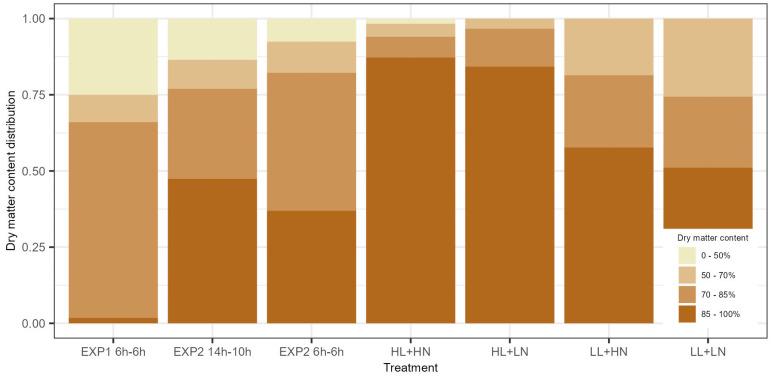
Average dry matter content distribution of the grains on an ear for all treatments in EXP1, EXP2, and EXP3. HL, LL, HN, and LN refer to ‘high light intensity’, ‘low light intensity’, ‘high nutrient concentration’, and ‘low nutrient concentration’, respectively. The dry matter contents, from dark to light brown, are >85%, 70–85%, 50–70%, and <50%.

Across all experiments, grain quality was observed through the grains’ average dry matter content per ear. In EXP1, the 6 h treatment resulted in less than 2% of the ears having grains with an average dry matter content of >85% (Figure 4), while in general, the maximum moisture levels for safe storage are set at 14% [21]. In EXP2, the dry matter content distribution improved. Comparable dry matter content distributions were observed for the grains in both treatments, with less than 50% of the ears having grains with an average dry matter content higher than 85%. A clear difference in dry matter content distribution was observed between the high- and low-light-intensity treatments in EXP3. In the high-light-intensity treatments in EXP3, more than 80% of the ears had grains with an average dry matter content higher than 85%, while this was only slightly more than 50% in the low-light-intensity treatments.

**Figure 5:**
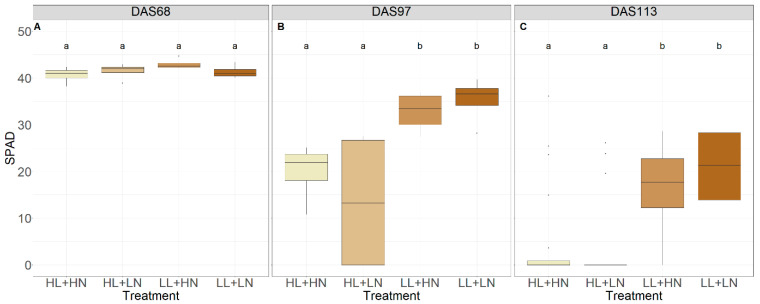
SPAD of flag leaves for all treatments in EXP3 on different days after sowing (DAS) during grain filling and ripening: 68 DAS (**A**) (grain filling), 97 DAS (**B**) (grain filling and ripening), and 113 DAS (**C**) (ripening). HL, LL, HN, and LN refer to ‘high light intensity’, ‘low light intensity’, ‘high nutrient concentration’, and ‘low nutrient concentration’, respectively. Different letters indicate statistical differences (*p* < 0.05) within the experiments.

Flag-leaf SPAD values, measured over three days during grain filling and ripening in EXP3, are shown in Figure 5. No significant difference in SPAD was observed between the treatments in EXP3 (*p* > 0.05, Figure 5A) on the day that the EC was lowered to 1000 µS cm^−1^ (68 DAS; grain filling). At 97 DAS (grain filling and ripening) (Figure 5B) and 113 DAS (ripening) (Figure 5C), SPAD values were significantly lower in the two high-light-intensity treatments in comparison with the low-light-intensity treatments (*p* < 0.05 and *p* < 0.001, respectively), where 97 DAS corresponds to the moment when the EC was lowered to 500 µS cm^−1^, and 113 DAS represents the time of harvest. At 113 DAS, most SPAD values for both high-light-intensity treatments reached 0, except for some outliers. 

The results of the kernel and wholemeal flour characterisation are presented in Table 3. In EXP3, the moisture content of the kernel and the thousand kernel weight values were lower for the high-light-intensity treatments in comparison with the low-light-intensity treatments. The HL + HN treatment showed the overall highest concentration in wet gluten (27.1 ± 0.2 g 100 g^−1^ wholemeal flour) and in dry gluten (9.2 ± 0.1 g 100 g^−1^ wholemeal flour), and a high protein concentration (13.5 g 100 g^−1^ dry matter). Only the 6 h treatment in EXP2 showed a higher protein concentration (14.1 g 100 g^−1^ dry matter), but no further analysis of wet and dry gluten could be performed due to the lack of sufficient material. Overall, the gluten index of all treatments in EXP3 was very high (>96.9%).

The HL + HN and HL + LN treatments in EXP3 showed intermediate falling numbers (respectively 154 ± 4 and 147 ± 3 s), whereas LL + HN showed the lowest value (101 ± 1 s) and LL + LN had the highest (176 ± 1 s). A lower value means that there is too much α-amylase activity in the flour to produce a good bread. More enzymatic activity results in a lower dough viscosity, with sticky dough as a result. In an ideal situation for bread baking, a falling number of 250–300 s is required [22]. The low falling numbers are reflected in low values of C3, C4, and C5 [23]. The lowest C3 value, derived from the Mixolab curve for the LL + HN treatment, indicates a lower peak viscosity and more starch degradation due to the higher α-amylase activity upon baking. Also, C4 and C5 were the lowest in the LL + HN treatment. 

The compositional data showed that grains in EXP2 contained more Cu and Zn compared to grains in EXP3, while grains in EXP3 contained the highest concentrations of Ca, Fe, and Mn. Within EXP2, Cu and Zn concentrations were higher in the 6 h treatment, while the concentrations of almost all other minerals were higher in the 14 h–10 h treatment. Zooming in on the differences between the treatments in EXP3, higher Fe and Mn concentrations were observed in LL + HN and HL + HN, whereas higher B, Ca, K, and Na concentrations were seen for HL + LN and HL + HN.

## 4. Discussion

### 4.1. Timing of Ear Emergence

Wheat is a long-day plant that requires a period of darkness shorter than a critical night length to induce flowering [8,24]. This explains the lack of ear emergence and flowering until 199 DAS in the 12 h treatment in EXP1, compared to this occurring two months after harvest (at 131 DAS) in the 6 h treatment. This absence of ear emergence under a 12 h light regime was also observed by Evtushenko and Chekurov [8] for four out of twelve spring wheat cultivars under low-light-intensity conditions. Although this issue was resolved under higher light intensities, ear emergence still took between 84.8 and 107.7 days for these wheat cultivars [8]. In another experiment on the wheat variety Servus using environmental conditions comparable to those in our EXP1, but with a 14 h–10 h light regime from the start of wheat development until maturity and with the plants being grown in soil, flag leaves emerged 79 DAS [25], or 13 days later than ear emergence, in a 6 h light regime. This demonstrates that a shorter light–dark cycle of 6 h can speed up the vegetative phase. Given these promising results, the 6 h light–dark cycle was used in all subsequent experiments, at least until the start of stem elongation.

The period from sowing until ear emergence in the 6 h treatment in EXP2 further decreased and was six days shorter (60 DAS) in comparison with EXP1 (66 DAS). This gain was caused by the higher light intensity used in EXP2. The timing of ear emergence under a 6 h light–dark cycle can hence be slightly improved by increasing the light intensity. This was also observed in a study by Evtushenko and Chekurov [8], under a 12 h light–dark cycle, where increasing the light intensity from 70 µmol m^−2^ s^−1^ to 360 µmol m^−2^ s^−1^ accelerated ear emergence by 15 days on average. 

After the initial growth for 37 days under the 6 h light–dark cycle, transition to a 14 h–10 h light regime around the start of stem elongation in EXP2 shortened the period until ear emergence even further, to 56 days, i.e., a gain of 10 days compared to EXP1. This timing seems relatively long in comparison with the 39–41 DAS that has been previously reported under speed-breeding conditions, wherein a fixed 22 h–2 h light–dark cycle was used during development for three wheat cultivars, including a 5-day germination period [6]. This difference could be partly explained by the longer day length and/or by the higher daytime temperature [26] of 22 °C used by Watson et al. [6] compared to this being 19.5 ± 1.9 °C in the 14 h–10 h treatment in our EXP2. For other wheat cultivars grown under speed-breeding conditions, a longer period of 63 days from sowing to ear emergence [6], and 46–50 days from sprouting to ear emergence [7], has been reported. These findings are comparable to the 56 days observed in EXP2’s 14 h–10 h treatment. However, it is important to note that our treatment involved a unique combination of two photoperiods, transitioning around stem elongation. This stage is recognised as critical for grain development in wheat [16]. Evtushenko and Chekurov [8] reported a range of 36 to 67 days from sowing until ear emergence for twelve spring wheat cultivars under a 16 h–8 h light regime with a light intensity of 360 µmol m^−2^ s^−1^. This shows that the timing of ear emergence is strongly cultivar-dependent and can be shortened by implementing a 6 h light–dark cycle and increasing the light intensity, although sensitivity to these parameters is also cultivar-dependent.

In EXP3, the period from sowing until ear emergence decreased to 51–54 days, which represents another gain of two to five days compared to the 14 h–10 h treatment in EXP2. This reduction might be linked to the slightly higher daytime temperature of 20.5 ± 1.4 °C in EXP3 compared to 19.5 ± 1.9 °C in EXP2 [26]. However, when comparing the timing of ear emergence, expressed in growing degrees, ears still emerged earlier in EXP3 (934–974 °Cd) compared to the 14 h–10 h treatment in EXP2 (1063 °Cd). This observation can thus also be attributed to the higher light intensities received by the growing plants, as the lamps in EXP3 remained fixed at a constant height. In EXP3, ear emergence in the HL treatments started three days earlier compared to the LL treatments. The minor differences in light intensity between the low- and high-light-intensity treatments in EXP3, as compared to the greater differences in intensity between EXP1 and the 6 h treatment in EXP2, are reflected in more modest differences in the timing of ear emergence. Consequently, the effect of light intensity on the timing of heading seems quantitative. 

### 4.2. Period of Ear Emergence to Harvest

In EXP2, new tillers were being formed until harvest, which resulted in many tillers without ears and tillers with non-fertile ears. In addition, non-synchronised grain maturation of tillers was observed. These observations indicate an imbalance in the nutrient supply, probably related to a high nitrogen availability [4]. In addition, the EC of the nutrient solution on the table, measured before adding fresh nutrient solution, increased after flowering, indicating a decrease in plant nutrient uptake. Since nitrogen is important for vegetative growth and an oversupply of nitrogen is known to delay senescence and nutrient redistribution [4,27], we decided to lower the nutrient solution’s EC, and, in turn, the nitrogen content, in EXP3 in two steps: (i) to 1000 µS cm^−1^ a few weeks after the start of flowering (68 DAS), and (ii) to 500 µS cm^−1^ around the end of the grain-filling stage (98 DAS). This lowering of the EC successfully resulted in the absence of new tiller formation during grain filling and ripening in all treatments in EXP3. The HL treatment promoted a relatively fast and synchronous maturation, characterised by a high percentage of ears with ripe grains, while low light intensity hindered this effect, despite minimal differences of three days in ear emergence. The mean stem flag-leaf SPAD reached a comparable maximum for all treatments at 68 DAS, 17 days after setting an equal light intensity for all treatments, which indicates a comparable initial flag-leaf development. When the EC was lowered to 1000 µS cm^−1^, flag-leaf SPAD values started to decline more sharply under the HL treatments in comparison with the LL treatments, which could be related to the higher number of shoots under HL and, therefore, the lower nitrogen availability per shoot. Therefore, a decrease in nitrogen availability under HL conditions could accelerate flag-leaf senescence and synchronise maturation [4,28,29].

Since the percentage of ears with ripe grains at harvest differs between treatments in all experiments, a comparison of the duration from ear emergence to harvest between the treatments is not relevant. However, based on the high percentage of ears with ripe grains under HL in EXP3, a minimum of about 60 days was required to go from ear emergence to ripe grains in all shoots. This is relatively long in comparison with speed-breeding conditions, where the complete growth cycle takes between 63 and 70 days [4 (70 d), 9 (65 d), 10 (70 d), 31 (63 d), 47 (67 d)]. Besides the longer day length under speed-breeding conditions, some other factors, like the temperature and plant density, may also contribute to the shorter period. Temperatures above 22 °C (Bugbee et al., 1994; Monje and Bugbee, 1998; Watson et al., 2018) [5,6,26] and plant densities higher than 700 plants per m^2^ [2,5,26] are frequently used under speed-breeding conditions. Higher temperatures are known to decrease the time taken until harvest. Bugbee et al. [26] observed a decrease in the total growth cycle duration of 12 days for a temperature increase from 19 °C to 22 °C for wheat grown in a closed environment.

A higher plant density is known to decrease the final number of tillers in wheat [30]. As such, the plant densities used in this study, of 225 to 275 plants per m², increased the number of tillers compared to speed-breeding conditions. For all 14 h–10 h treatments in EXP2 and EXP3, an increase in ear ripeness with tiller age was observed. Consequently, a higher number of tillers will increase the length of the pre-harvest period. Increasing the plant density to accelerate ripening seems promising. Nevertheless, ear-bearing tillers contribute to yield. As such, the trade-off between greater yield and faster ripening should be kept in mind for future studies in searching for an optimal plant density for controlled environment conditions. 

### 4.3. Yield

The total dry weight of grains per plant almost doubled by changing the light–dark cycle from 6 h to 14 h–10 h at the beginning of stem elongation in EXP2, although the daily light integral only increased by 17%. This increase in yield cannot be solely attributed to the higher daily light integral, indicating that the short cycle during generative development in our study was suboptimal for optimizing yield. While the total number of shoots per plant and the dry weight per grain did not significantly differ between both treatments in EXP2, the change in light regime to 14 h–10 h around the beginning of stem elongation triggered ear formation and development, almost doubling the number of shoots with grains and increasing the number of grains per shoot. The use of a shorter photoperiod has, however, been demonstrated to extend the duration of the stem-elongation phase, thereby enhancing the availability of assimilates crucial for ear growth. Prior studies have shown how this extension positively impacts yield by increasing both the number of ear-bearing tillers and the number of grains per ear [16]. However, it must be noted that González et al. [16] compared an average natural photoperiod of 13.4 h during the stem-elongation phase with a longer 19.4 h photoperiod. In our study, the longer photoperiod (14 h), applied during stem elongation, shortened the duration of stem elongation compared to the much shorter photoperiod of 6 h. Although the stem-elongation period was shorter, the 14 h light treatment resulted in a higher yield, evidenced by a higher number of ear-bearing shoots and grains per ear. It is probable that the 14 h photoperiod facilitated a greater supply of assimilates to the ear compared to the twice-repeated 6 h photoperiods. 

Inspired by the findings in EXP2, we replicated the combination of a 6 h light–dark cycle transitioning to a 14 h–10 h light–dark cycle around stem elongation in EXP3. This allowed us to further investigate the impact of light intensity and nutrient concentration. The total number of shoots and number of shoots with grains increased with light intensity, which is consistent with the literature [9,30]. The number of grains per ear and the dry weight per grain are determined during the generative phase and are expected to also increase with light intensity [9,16,31], but the opposite was observed in our study. Because the light intensity was lowered to LL levels in the two HL treatments in EXP3 shortly after ear emergence to avoid extreme temperatures for the ears growing close to the lamps, a direct effect of light intensity was expected in the period before ear emergence. It is known that the floret number and size, as determined during the stem-elongation phase, play an important role in the final grain number and size. As such, the eventual grain yield potential is determined during the stem-elongation phase and depends on its duration [16,32]. 

However, the relatively small difference in the stem-elongation duration of at maximum a few days hints at another explanation for the observed decrease in the number of grains per ear and dry weight per grain with the increased light intensity, bringing us back to the period after the light intensity was lowered and equalised between all treatments. Frequently, an increase in plant density is accompanied by an increase in the number of ears per area [33,34,35], while both the thousand kernel weight and the number of kernels per ear decrease. This decrease is linked to increased competition between the plants, caused by the increased ear density [33,35,36]. Since the number of shoots with ears increased under high light intensity, and grain filling and ripening occurred under the same (forced) light intensity in all treatments, an increased competition in the HL treatments might explain the lower dry weight per kernel and number of kernels per ear. This increased competition is further confirmed by the faster leaf senescence in the HL plants compared to the LL plants following the decrease in EC to 1000 µS cm^−1^. Since flag-leaf photosynthesis plays an important role in grain filling [37], faster flag-leaf senescence eventually results in less assimilate availability during grain filling [38,39,40].

In all 14 h–10 h treatments, after initial growth under 6 h light–dark cycles until stem elongation (EXP2 and EXP3), a yield between 1700 and 2400 g m^−2^ was obtained under a light intensity of approximately 600 µmol m^−2^ s^−1^ during grain filling and ripening. Bugbee et al. [26] and Dong et al. [2,3] observed a grain yield between 400 and 1050 g m^−2^ under a comparable light intensity of 500–800 µmol m^−2^ s^−1^ but a photoperiod of at least 20 h. Although only 70 days were needed until harvest in these studies [2,3,26], the yield is relatively low compared to that of the 6 h light–dark cycle followed by the 14 h–10 h cycle used in our EXP2 and EXP3. In their study, Yunze and Shuangsheng [7] observed a decrease in growth cycle duration from 126 to 88 days with an increase in photoperiod from 12 to 24 h, but this was coupled with a decrease in the grain yield from 2104 to 1347 g m^−2^. Monje and Bugbee [5] observed relatively high average grain yields of 1397 and 1583 g m^−2^, dependent on atmospheric CO_2_ concentration, within 70 days under a 20 h photoperiod. However, the high light intensity of 1400 µmol m^−2^ s^−1^ in Monje and Bugbee [5]’s study drastically decreased the energy efficiency compared to other research, including our innovative combination of 6 h cycles followed by a 14 h–10 h light–dark treatment employed in EXP2 and EXP3. In future plant-growing systems, energy efficiency should be prioritised, and exploring lower light intensities that still promote optimal grain development and yield can improve energy efficiency and economic viability. Although the use of different wheat varieties, planting distances, and microclimate settings in the literature makes an exact comparison between growth strategies challenging, the 6 h light–dark cycle followed by a 14 h–10 h light–dark cycle after the beginning of stem elongation seems a promising option for boosting wheat production. A targeted increase in photoperiod after flowering [7] or a decrease in light intensity at the seedling stage [2] could potentially further increase energy efficiency. These observations indicate that future research needs to determine an optimal balance between the photoperiod, light intensity, and light regime tailored to different wheat varieties and different developmental stages, thereby achieving higher yields and energy-efficient production.

### 4.4. Quality

In the context of future plant-growing systems, it is crucial to not only focus on grain yield but also consider grain quality, and flour quality and composition. The average grain dry matter content of the ears, and thus the storability of the grains, increased over our experiments, showing that this trait can be steered with light intensity.

In EXP2, the concentrations of most minerals were in line with those reported in the literature [41,42,43], except for B, Cu, and Zn, which showed concentrations nearly twice as high as the values reported in the literature, while the concentration of Fe was relatively low. The concentrations of most minerals were notably elevated in all EXP3 treatments compared to the values reported in the literature. Particularly, the concentration of B exceeded double the typically observed values. The elevated concentrations of most minerals might be related to the immediate availability of the nutrients in the hydroponic system. The high concentrations of B, Cu, and Zn might have resulted from the release of these minerals from the lava-rock substrate [44]. While the precise cause of variations both between and within the experiments remains elusive, differences between the experiments may be attributed to differences in the nutrient solutions, irrigation methods, physical parameters of the growing system influencing root mineral uptake, and environmental conditions, whereas differences within the experiments should be attributed to the applied treatments. 

When evaluating baking-quality-related parameters, such as the protein content and gluten index, favourable baking characteristics of the wholemeal flours were measured. However, the consistently lower falling numbers observed across all treatments in EXP3 would currently inform rejection of these wheat batches prior to milling. Pre-harvest sprouting is one of the major causes of a low falling number (Newberry et al., 2018) [12]. Resistance to pre-harvest sprouting is believed to be predominantly determined by seed dormancy [13,45]. Since the wheat variety Servus scores high on the falling-number criterion of Saaten-Union, a relatively low susceptibility to pre-harvest sprouting is expected (A. Boenisch, Saaten-Union, Germany, ‘pers. com.’). No visible signs of pre-harvest sprouting were observed in the experiments, and rain, the main environmental factor affecting pre-harvest sprouting [13,46,47], was absent in the closed controlled environments, mimicking plant-growing systems on Mars. Therefore, pre-harvest sprouting is presumably not the cause of the lower falling numbers in all EXP3 treatments. Another significant factor that can lead to a low falling number is the presence of late-maturity α-amylase, which involves α-amylase synthesis at a later stage of grain development and is partially determined by genotype [13,48]. Late-maturity α-amylase is typically associated with temperature shocks or prolonged cold periods during specific stages of grain development [13,49,50], neither of which occurred in EXP3, where a stable daytime temperature of 20.5 ± 1.4 °C was maintained. Despite decades of research, late-maturity α-amylase remains a phenomenon not yet fully understood [49]. Currently, a low falling number leads to the rejection of the grain for bread baking. However, recent evidence suggests that the impact of late-maturity α-amylase on end-product quality differs from the effects of pre-harvest sprouting [11,12], although this debate is still ongoing [51]. A less common cause of low falling numbers is retained pericarp α-amylase, where low temperatures inhibit the degradation of α-amylase in the pericarp [48]. Since the growth chamber in EXP3 never reached low temperatures, retained pericarp α-amylase cannot account for the observed low falling numbers. 

As the falling number tends to increase with higher nitrogen fertilisation [52,53], although occasionally, opposite trends have been observed [54], the abrupt change in nutrient concentration at 68 and 98 DAS provides a potential explanation for the low falling numbers observed in EXP3. Nevertheless, the limited data do not permit us to draw final conclusions about the precise cause of these low falling numbers, but they do suggest an interesting avenue for further research. 

These findings emphasise the need to consider not only grain yield and quality, but also parameters related to baking and the nutritional quality of the flour. Surprisingly, the falling number and other baking-quality-related parameters are often overlooked in most studies focusing on wheat cultivation in controlled environments. The importance of investigating the potential impact of intensive indoor wheat cultivation on nutritional value and baking quality has been emphasised by Asseng et al. [1]. Collaboration between researchers and wheat breeders can facilitate the identification of wheat varieties with improved resistance to pre-harvest sprouting and late-maturity α-amylase, ultimately enhancing baking quality and reducing the occurrence of low falling numbers.

## 5. Conclusions

In conclusion, the implementation of a 6 h light–dark cycle in a prospective plant-growing system, whether for use on Earth or in space, facilitates ear emergence and the induction of flowering in long-day spring wheat plants, even with a relatively low daily light integral. The timing of ear emergence under a 6 h light–dark cycle was comparable to speed-breeding conditions, and could be accelerated by increasing the photoperiod around stem elongation and increasing the light intensity. This approach hence holds potential to enhance energy efficiency and economic viability for the cultivation of wheat in controlled environments. 

Maintaining a consistent 6 h light–dark cycle during the generative phase did not lead to maximum yield. However, transitioning the light regime to a longer photoperiod of 14 h–10 h light–dark around the onset of stem elongation significantly increased wheat production. This strategy offers a promising avenue to boost production levels. Future efforts should again be directed towards striking the right balance between photoperiod and light intensity at various developmental stages. Other environmental factors like the N supply and plant density can be specifically optimised for controlled growing conditions to achieve synchronous maturation. 

Despite the controlled microclimate and the absence of rain in a closed environment, lower falling numbers were observed without a clear cause. This emphasises the need to not only consider grain yield, but also the quality and composition of the flour in controlled-environment experiments. Investigating the interaction between genotype and environmental factors, as well as the influence of nutrient concentrations and other variables, is essential to understand and mitigate the occurrence of low falling numbers and could ultimately lead to the production of flours with superior baking quality.

## Figures and Tables

**Table 1:** Environmental settings and variables with standard deviation for all treatments in EXP1, EXP2, and EXP3: light regime before and after the start of stem elongation; average measured electrical conductivity (EC, µS cm^−1^), pH, and dissolved oxygen (DO, mg L^−1^) of the nutrient solution on the growth table (in EXP3: before lowering the nutrient concentration); light intensity (µmol m^−2^ s^−1^); daytime air temperature (°C); nighttime air temperature (°C); relative humidity of the air (%); atmospheric CO_2_ concentration (ppm).

	EXP1		EXP2		EXP3			
	6 h–6 h	12 h–12 h	6 h–6 h	14 h–10 h	HL + HN	HL + LN	LL + HN	LL + LN
Light–dark cycle before start of stem elongation	6 h–6 h	12 h–12 h	6 h–6 h	6 h–6 h	6 h–6 h	6 h–6 h	6 h–6 h	6 h–6 h
Light–dark cycle after start of stem elongation	6 h–6 h	12 h–12 h	6 h–6 h	14 h–10 h	14 h–10 h	14 h–10 h	14 h–10 h	14 h–10 h
Light intensity (µmol m^−2^ s^−1^)	93 ± 41 ^1^	93 ± 41 ^1^	440–720 ^2^	440–720 ^2^	524 ± 12 ^1^	520 ± 20 ^1^	318 ± 15 ^1^	310 ± 14 ^1^
EC (µS cm^−1^)	2142 ± 87	2169 ± 232	2010 ± 178	2030 ± 195	1954 ± 28	1569 ± 34	1960 ± 47	1548 ± 29
pH	7.2 ± 0.3	7.5 ± 0.3	6.6 ± 0.4	6.6 ± 0.3	6.5 ± 0.1	6.5 ± 0.1	6.4 ± 0.1	6.5 ± 0.1
DO (mg L^−1^)	NA	NA	3.50 ± 1.78	3.41 ± 1.69	5.60 ± 0.16	5.41 ± 0.12	5.47 ± 0.11	5.31 ± 0.03
Daytime temperature (°C)	20.0 ± 0.6	20.0 ± 0.7	20.6 ± 0.8	19.5 ± 1.9	20.5 ± 1.4			
Nighttime temperature (°C)	17.7 ± 0.7	17.8 ± 0.6	17.1 ± 0.8	18.0 ± 1.6	17.9 ± 1.1			
Relative humidity (%)	73.1 ± 10.0	73.1 ± 11.2	84.1 ± 7.9	81.2 ± 8.8	61.0 ± 6.2			
CO_2_ concentration (ppm)	485 ± 97	473 ± 75	699 ± 72	693 ± 74	745 ± 223			

^1^ Light intensity at table level; ^2^ range of light intensities at canopy level. Abbreviations: HL = ‘high light intensity’, LL = ‘low light intensity’, HN = ‘high nutrient concentration’, and LN = ‘low nutrient concentration’.

**Table 2:** Timings (measured in days after sowing) of cultivation measures, ear emergence, and harvest for the 6 h treatment in EXP1 and all treatments in EXP2 and EXP3. Abbreviations: HL = ‘high light intensity’, LL = ‘low light intensity’, HN = ‘high nutrient concentration’, and LN = ‘low nutrient concentration’.

	EXP1	EXP2		EXP3			
	6 h–6 h	6 h–6 h	14 h–10 h	HL + HN	HL + LN	LL + HN	LL + LN
Change to 14 h–10 h light regime			38	37	37	37	37
Start of ear emergence	66	60	56	51	51	54	54
Lowering of light intensity				51	51		
Lowering of EC to 60%		116	116				
Lowering of EC to 50%				68	68	68	68
Lowering of EC to 25%				98	98	98	98
Start of harvest	131	122	127	111	111	111	111
End of harvest	138	126	136	113	113	119	119

**Table 3:** Kernel and wholemeal flour characteristics for all treatments in EXP3 and, where feasible, EXP2: moisture content of the kernel (MC, g 100 g^−1^); test weight (TW, kg hL^−1^); thousand kernel weight (TKW, g); dry matter content of wholemeal flour (g 100 g^−1^); ash content (g 100 g^−1^); protein content (g 100 g^−1^ DM); wet gluten (WG, g 100 g^−1^ flour); dry gluten (DG, g 100 g^−1^ flour); gluten index (GI, %); falling number (FN, s); compositional attributes (mg kg^−1^ wholemeal flour): boron (B), calcium (Ca), copper (Cu), iron (Fe), potassium (K), magnesium (Mg), manganese (Mn), sodium (Na), phosphorus (P), and zinc (Zn); and Mixolab dough properties: water absorption (WA, %) and C1–C5. Abbreviations: HL = ‘high light intensity’, LL = ‘low light intensity’, HN = ‘high nutrient concentration’, and LN = ‘low nutrient concentration’.

	Kernel			Wholemeal Flour						
	MC	TW	TKW	DM	Ash	Protein	WG *	DG	GI	FN
	g 100 g^−1^	kg hL^−1^	g	g 100 g^−1^	g 100 g^−1^	g 100 g^−1^ DM	g 100 g^−1^ Flour	g 100 g^−1^ Flour	%	s
EXP2 6 h–6 h	-	-	51.1 ± 1.2 ^a^	87.5 ± 0.2 ^a^	1.9 ± 0.2 ^a^	14.1	-	-	-	-
EXP2 14 h–10 h	-	-	48.6 ± 0.7 ^ab^	87.5 ± 0.2 ^a^	2.3 ± 0.2 ^a^	13.0	-	-	-	-
EXP3 HL + HN	12.57 ± 0.01 ^b^	75.2 ± 0.1 ^b^	42.4 ± 1.7 ^b^	89.1 ± 0.2 ^b^	2.5 ± 0.2 ^a^	13.5	27.1 ± 0.2 ^b^	9.2 ± 0.1 ^b^	97.5 ± 0.7 ^a^	154 ± 4 ^b^
EXP3 LL + HN	13.50 ± 0.01 ^a^	77.2 ± 0.1 ^a^	46.7 ± 2.0 ^ab^	88.3 ± 0.2 ^ab^	2.3 ± 0.2 ^a^	13.0	25.7 ± 0.5 ^a^	8.7 ± 0.0 ^a^	96.9 ± 0.8 ^a^	101 ± 1 ^a^
EXP3 HL + LN	12.39 ± 0.04 ^b^	77.2 ± 0.3 ^a^	43.6 ± 1.2 ^b^	89.2 ± 0.2 ^b^	2.4 ± 0.2 ^a^	13.2	25.6 ± 0.3 ^a^	8.8 ± 0.2 ^ab^	98.3 ± 0.2 ^a^	147 ± 3 ^b^
EXP3 LL + LN	13.56 ± 0.04 ^a^	77.7 ± 0.1 ^a^	47.1 ± 1.3 ^ab^	88.4 ± 0.2 ^ab^	2.3 ± 0.2 ^a^	13.1	26.2 ± 0.4 ^ab^	8.9 ± 0.0 ^ab^	98.1 ± 0.6 ^a^	176 ± 1 ^c^
	**B** **mg kg^−1^**	**Ca** **mg kg^−1^**	**Cu** **mg kg^−1^**	**Fe** **mg kg^−1^**	**K** **mg kg^−1^**	**Mg** **mg kg^−1^**	**Mn** **mg kg^−1^**	**Na** **mg kg^−1^**	**P** **mg kg^−1^**	**Zn** **mg kg^−1^**
EXP2 6 h–6 h	4.46	385	12.3	20.9	4650	1370	46.3	29.3	3820	191
EXP2 14 h–10 h	4.59	362	10.3	25.2	5770	1460	49.0	35.7	4200	133
EXP3 HL + HN	5.47	485	6.66	34.3	6280	1550	65.6	37.8	4540	49.7
EXP3 LL + HN	4.46	459	5.84	35.9	5870	1510	68.5	33.8	4410	48.3
EXP3 HL + LN	4.88	491	7.56	32.7	6130	1620	55.4	37.5	4540	49.5
EXP3 LL + LN	4.33	445	7.79	32.9	5650	1520	55.0	32.6	4320	45.4
	WA *	**C1**		**C2**		**C3**		**C4**		**C5**
	**%**	**Time** **(min)**	**Torque** **(Nm)**	**Time** **(min)**	**Torque (Nm)**	**Time (min)**	**Torque (Nm)**	**Time** **(min)**	**Torque (Nm)**	**Torque (Nm)**
EXP3 HL + HN	65.0	5.2	1.141	17.5	0.342	22.3	1.196	32.2	0.431	0.764
EXP3 LL + HN	62.0	4.8	1.137	17.7	0.334	21.8	1.055	33.2	0.248	0.447
EXP3 HL + LN	62.0	4.8	1.149	17.8	0.339	22.4	1.245	33.7	0.436	0.780
EXP3 LL + LN	62.9	5.1	1.090	17.3	0.343	21.9	1.220	33.4	0.414	0.763

* Corrected for 14% moisture content in wholemeal flour. ^a–c^ Values followed by different letters differ significantly (*p* < 0.05).

## Data Availability

The original contributions presented in the study are included in the article/Supplementary Materials, further inquiries can be directed to the corresponding author.

## Supplementary Materials

The following supporting information can be downloaded at https://www.mdpi.com/article/10.3390/foods13050750/s1: Figure S1: The light spectra used in experiment one (EXP1), experiment two (EXP2), and experiment three (EXP3); Table S1: Average measured electrical conductivity (EC, µS cm^−1^), pH, and dissolved oxygen (DO, mg L^−1^) of the nutrient solution on the growth table, measured before and after adding new solution to the table. Within light–dark treatments, statistical differences between mean values of these parameters, before and after adding fresh nutrient solution, were tested. Statistical differences between the mean values of EC, pH, and DO were tested using a student’s t-test. Different letters indicate significant differences (*p* < 0.05).
