# Supplementary material for: The Impact of a Six-Hour Light–Dark Cycle on Wheat Ear Emergence, Grain Yield, and Flour Quality in Future Plant-Growing Systems
Source: Foods. 2024 Feb 28;13(5):750. doi: 10.3390/foods13050750 (PMC10931310; doi:10.3390/foods13050750)
Supplement: Supplementary file 1 [file foods-13-00750-s001.zip › Supplementary_File.pdf]

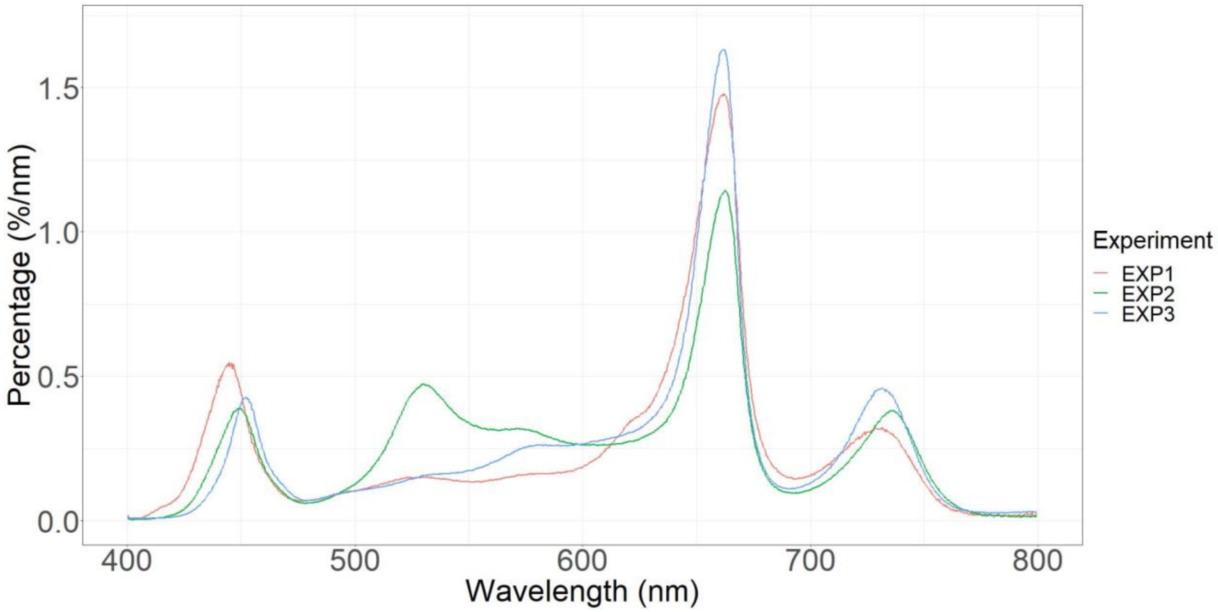

Figure S1. The light spectra used in experiment 1 (EXP1), experiment 2 (EXP2) and experiment 3 (EXP3).

Table S1. Average measured electrical conductivity (EC, µS cm^{-1}), pH and dissolved oxygen (DO, mg L^{-1}) of the nutrient solution on the growth table, measured before and after adding new solution to the table. Within light-dark treatments, statistical differences between mean values of these parameters, before and after adding fresh nutrient solution were tested. Statistical differences between the mean values of EC, pH and DO were tested using a student's t-test. Different letters indicate significant differences ($P < 0.05$).

	6h-6h		14h-10h	
	Before	After	Before	After
EC (µS cm^{-1})	2027 ± 336[a]	1925 ± 235[a]	2117 ± 370[a]	1952 ± 224[a]
pH	6.7 ± 0.3[a]	6.6 ± 0.4[a]	6.7 ± 0.1[a]	6.6 ± 0.3[b]
DO (mg L^{-1})	1.46 ± 0.55[a]	4.05 ± 0.92[b]	1.18 ± 0.19[a]	3.67 ± 0.85[b]
